# Review of the Current Knowledge of Reactive Attachment Disorder

**DOI:** 10.7759/cureus.31318

**Published:** 2022-11-10

**Authors:** Neha Irfan, Arun Nair, Jessica Bhaskaran, Maksuda Akter, Tabitha Watts

**Affiliations:** 1 School of Medicine, Royal College of Surgeons in Ireland - Medical University of Bahrain, Muharraq, BHR; 2 Pediatrics, Saint Peter’s University Hospital, Somerset, USA; 3 School of Medicine, American International Medical University, Gros Islet, LCA; 4 Pediatric Emergency Medicine, John H. Stroger, Jr. Hospital of Cook County, Chicago, USA

**Keywords:** dynamic model of the insecure cycle, attachment theory, current mediation hypothesis, depression, pediatrics, reactive attachment disorder

## Abstract

Reactive attachment disorder (RAD), classified under Trauma and Stressor Related Disorders in the DSM-5 manual, is a childhood psychiatric illness due to familial or social neglect or due to maltreatment. It is characterized by an inhibited and withdrawn social and emotional behavior toward an adult caregiver, typically before the age of 5. Neurobiological changes in patients with RAD have been shown to be substantially significant with features such as loss of grey matter volume and neurotransmitter deficiencies that not only impact the ability to form healthy attachments but also increase the risk of comorbidities such as depression and anxiety.

Different theories, including the current mediation hypothesis and learning theory of attachment, showed childhood maltreatment from caregivers and desensitization toward deficiencies in social development in children from special education teachers to be key components in the development of RAD. Patients with RAD had an increased risk of developing psychiatric comorbidities, including learning disabilities and mood disorders. Institutionalized care and childhood maltreatment have a significant impact on the development of RAD.

RAD is an underdiagnosed and underreported condition with significant repercussions that can severely impact the development of a child. By being able to raise awareness and promote further research into refining the diagnostic methodology, treatment protocols, and long-term follow-up, children afflicted with this condition may be able to develop better socio-emotional bonds and reduce the incidence of comorbidities such as depression and attention deficit hyperactivity disorder.

## Introduction and background

Reactive attachment disorder (RAD) is a childhood psychiatric disorder described as a condition affecting social functioning in children. It is characterized by either an inhibited or disinhibited subtype, and symptoms for both vary [1]. The inhibited subtype is characterized by a cautious and highly watchful individual, whereas the disinhibited subtype typically presents with an overly friendly child with engagement to strangers with ease and a lack of desire to remain with the primary caregiver [1]. Risk factors that have been described as influential in the development of RAD include child abuse and neglect, as well as institutionalized care [1]. The Diagnostic and Statistical Manual 5th Edition (DSM-5) classifies RAD under trauma and stressor-related conditions of early childhood, and it is usually identified in children younger than five years who present with symptoms of internalizing behavior such as failure to seek comfort, lack of positive effect, and hypervigilant states without the presence of danger [1]. The prevalence of RAD is 1-2% in the general population [2]; however, true prevalence may vary in different regions of the world.

Institutionalized care puts children at a greater risk of emotional or physical abuse that is quantifiable as neglect and maltreatment, leading to negative health and development outcomes. During 2001-2018, it was estimated that the number of children brought up in congregate care ranges from 3.18 to 9.42 million [3]. The presence of structural neglect among institutionalized children can be attributed to inadequate resources such as staffing and unequal socio-emotional interaction between the child and their caregiver within the institutions [4]. Additionally, such institutions often suffer from depleted resources such as low numbers in staffing and therefore inadequate socio-emotional interactions [4]. The consequential developmental delay encompassing mind and body in children separated from family environments was first documented around 100 years ago and has been seldom studied then onward. Since then, much research has been conducted to study its effects including psychosocial and emotional disturbances. Reasons that a child may be placed in institutionalized care include poverty, orphaning, victims of abuse, and even mental illness.

Several studies addressed the effect of institutionalization on attachment patterns using the strange situation procedure (SSP) [1], which presented high rates of insecure attachment of the disorganized type. Maltreatment or neglect during the first three years of life increases the risk of attachment disorders such as RAD and disinhibited social engagement disorder (DSED) in children reared in this setting. RAD affects both the physical and mental health of the developing individual, which is why an interdisciplinary approach is required for its diagnosis and management. There is no specific diagnostic method available for RAD; hence, a series of tests such as the SSP, the attachment formation rating scale, and the rating for Inhibited attachment disorder [5] are used. However, the focus of RAD in these tests is lower than that of DSED, which further encourages the multiple testing systems for its diagnosis.

The deficits that have been documented span multiple areas including physical growth such as low height, weight, and head circumference, and cognitive function such as IQ or development quotient (DQ) for infants. The effect of institutionalization seemed most pronounced when the children have the least access to individualized caregiving coinciding with deprivation during the early developmental sensitive periods. The neurobiological implications of RAD are still very under-researched, but some studies have proposed that children with RAD are more likely to have multiple comorbidities such as attention deficit hyperactivity disorder (ADHD; 52%), post-traumatic stress disorder (PTSD; 19%), and autism spectrum disorder (14%) [1].

This article aims to further explore and understand the existing theories relating to the development of RAD, the neurobiological changes that occur in children affected, and the current recommendations in treatment for this condition.

## Review

Neurobiological changes in RAD

Neurobiological changes in RAD have been seldom examined in the existing literature. While understanding the importance of the relationship of the attachment between the child and their primary caregiver, deeper comprehension of the neurobiological changes that occur due to this disorder can aid in better and more targeted treatment through both non-pharmacological and pharmacological modalities.

It has been noted that early childhood experiences can affect the structure of neurons as well as the communication between them [6]. If altered due to disease pathology, this can have astounding effects on the development of different regions of the brain, especially the connections between the emotional regions and the different cortices that aid in shaping one’s personality and other higher functions. One of the primary issues that are associated with RAD is the affection and nurture between a caregiver and a child being diminished. A lot of factors play a role in this interaction such as basic needs including a sense of safety as well as good, but also a child’s physiological needs such as thermoregulation. This interaction is key in the development of the child’s sensory stimulation as it has been shown that the presence of a caregiver can reduce stress levels in a child when they are encountering a stressful situation.

A study by Newman et al. [7] stated that early disruptions in the attachment system can lead to a detrimental development of attachment when a person reaches adulthood. Such ramifications are concerning as this can lead to the development of other psychiatric disorders. Interestingly, Newman et al. mentioned that children who have had a history of maltreatment in their early childhood showed decreased intracranial volumes and a decreased size of the corpus callosum. When correlating with the neurobiological changes, a study by Reite et al. in 2010 described the changes in brain volume in different psychiatric disorders and found that both schizophrenia and bipolar disorder also had a slightly decreased total brain volume to intracranial volume ratio [8].

Returning to the complex interaction that is attachment, Chambers in 2017 [9] explored the interaction between the developing hypothalamus and levels of cortisol in developing infants. It was shown that oxytocin, which is a hormone produced during maternal bonding with their child, has been shown to decrease cortisol levels, especially in social interactions. In the instance where there is a decreased interaction between a mother and her infant, studies have shown that cortisol levels increase. This is significant as the hyperactivity of the hypothalamic-pituitary (HPA) axis from a lack of oxytocin significantly increases cortisol levels to the point where it is neurotoxic. These effects have been described to be long-lasting. Increased cortisol levels due to HPA hyperactivity have also been described in generalized anxiety disorder [10] in adults, and this would be a potential point of concern as long-term morbidity in patients who have been diagnosed with RAD.

Makita et al. in 2020 explored the cerebral changes in children diagnosed with RAD and found significant changes to the structure of the brain in these patients [11]. This study particularly described changes in the white matter tracts, especially within the corpus callosum and corticolimbic circuits a well. It was also reported that RAD was directly associated with a reduction in grey matter volume, especially in the visual cortex, and a change in the function of the ventral striatum. Makita et al., through examining the microstructure of white matter tract organization, found higher fractional anisotropy within the neuronal projections in the thalamic region. It was further concluded that elevated fractional anisotropy can be reflective of pathology within the brain and there have been several reports that have sustained this statement. It has been shown that patients who have a history of childhood maltreatment and have interrupted emotional regulation have been shown to have increased fractional anisotropy.

Children who have been placed in foster care tend to have an increase in the need to use mental health services [12]. It was also described that interrupted socio-emotional relationships can lead to the development of aggressive and hyperactive behavioral issues. A study by Weinberg in 2010 supports this statement [13], in that due to a combination of the impairment in the attachment system as well as neurotransmitter deficiencies, pharmacological interventions can treat comorbid conditions associated with attachment disorders such as RAD. These comorbid conditions include depression, ADHD, and PTSD; however, further research is required to substantiate and prove the efficacy of pharmacological treatment in attachment disorders. 

Exploration of theories regarding development of RAD

Existing Hypothesis and Etiological Propositions

The etiology of RAD has been investigated to a certain extent, according to our search of existing literature. Like many psychiatric diagnoses, RAD can be presumed to be multifactorial; however, some etiologies stand out as a more likely trigger for RAD. A form of trauma has consistently been described in the pre-existing literature. According to Ellis et al. [2], examples of predisposing trauma include severe emotional neglect in settings such as foster care, orphanages with a poor caretaker-to-child ratio, and in cases of parents who may be suffering from a mental or physical illness. The absence of emotional nurture and interaction has been described as resulting in poor language communication, cognitive impairment, and behavioral disorders.

Current Mediation Hypothesis

The current mediation hypothesis aims to discuss the relationship between a lack of emotional nurture and the development of RAD. This was described in a study by Cuyvers et al. [14], which explored the relationship between the symptoms of RAD and insecure attachment. Parental factors were assessed and included attributes such as lack of support, lengthy periods of isolation between the parent and child, and mood disorders such as depression. These characteristics were suggested to translate into more severe symptoms of RAD in their children. Children with such symptoms displayed an aberrant trust relationship with their caregivers which in turn reflected in a weak attachment with them. However, Cuyvers et al. also discussed conflicting results and theories with this hypothesis, such as studies conducted by Green et al. and Schroder et al. [15,16]. These studies described a null association between the degree of attachment between caregivers and children and the occurrence and severity of RAD symptoms. Green et al. suggested that behavioral issues were more likely to be an etiological input into the development of RAD compared to attachment issues.

The contrasting evidence concerning this hypothesis furthermore highlights the multifactorial quality of RAD. When investigating the etiology of RAD, it is important to carefully consider all angles of risk factors. A study conducted by Zeanah and Gleason in 2015 [17] described the potential causes of RAD and divided them between the environment in which the child is being raised and the factors that involve the vulnerability of the child. Poor home environments were commonly attributed to foster care and institutionalized care as well. There is a possible association between the degree of how severely impaired the caregiving environment is and how likely a child is to develop RAD; however, this may be very difficult to quantify with statistically significant results due to the multifactorial and qualitative nature of this condition. In support of the current mediation hypothesis, we found that Zeanah and Gleason mentioned two studies, conducted by Lyons-Ruth et al. [18] and Oliviera et al. [19] in 2009 and 2012, respectively, that described the relationship between the psychiatric profile and emotional interactions of the mother toward the child influenced indiscriminate behavior, which is defined as a “lack of selectivity in the choice of attachment figures” [18]. Furthermore, a national pregnancy survey conducted during the 1990s suggested that teenage mothers and drug use during and after pregnancy seemed to increase the likelihood of increasing the rates of RAD [5].

Child vulnerability factors included the caregiving environment they were placed in, which reflects well within the current mediation hypothesis. Studies have shown that factors such as adoption and institutionalized care, in conjunction with biological factors such as genetic diseases such as Williams syndrome, seem to have a partial role in the development of symptoms of RAD and DSED. Zeanah and Gleason also suggested that symptoms of RAD are correlated with the lack of attachment to certain caregiving figures and that enhanced caregiving seems to better the outcomes of RAD, also reflected in a study conducted by Gleason et al. [20].

Attachment Theory

The attachment theory, described in an article published by Bosmans et al. in 2020 [21] examined a correlation between attachment and security that a child may have with their caregiver and suggested that through an increase in attachment, a child may feel more secure and vice versa. It was also suggested that this model and the relationship between attachment and security can constantly remodel during life, but a general assumption was accepted that there is a consistent development of both variables by the age of 3. Although teachers are not the primary caregivers in many children’s lives, a sense of attachment and security can be attributed to this relationship, albeit temporary. It was described that children with a decreased attachment to their primary caregivers were shown to develop an overdependent behavior toward their teachers; however, teachers were found to have an underwhelming relationship with the affected child.

A possible insight into this theory is the sense of balance in the mental growth of children that is required to create a plausible and sustainable “caregiving figure” in their lives. Where this is a lack of a maintainable attachment with a primary caregiver, an overcompensation can occur with the next available attachment figure, which, in many cases, can be a teacher. This highlights the importance of an awareness of this condition not only among physicians but also among secondary caregivers such as teachers. Through this understanding, a more secure relationship with their primary caregiver can be initiated and an earlier intervention can be planned, thus improving outcomes for children affected with RAD.

Dynamic Model of the Insecure Cycle

In 2019, Kobak and Bosman [21] explored the connection between an innate attachment system in children that needs to be nurtured, especially during times of distress. A key point to note in this theory is that once learned, it is a permanent fixture for a child. This theory is crucial to implement in the potential genesis of RAD in our opinion as not only does it incorporate the importance of positive and negative events in life that can modify the innate attachment system.

Through an interconnection between the memories, new knowledge gained through life experiences, and coping mechanisms, it can be postulated that a child’s innate attachment system is very fluid, especially in early development. Kobak and Bosman suggested that memories can be replaced by new knowledge and can potentially aid in the development of coping mechanisms to prevent old memories from resurfacing. Negative memories associated with attachment can be further enhanced as the child developed in an episodic fashion, for example, when a caregiver’s response to a parenting situation involving the child may be negative and stressful to the child, this cycle can result in a negative interpretation of the caregiver’s behavior and therefore the child may begin to use coping mechanisms to deal with the negatively interpreted behavior toward them. Due to this vicious cycle, children may not be able to initiate support-seeking behavior and may end up using maladaptive behavioral techniques. This, unfortunately, can also result in a caregiver misinterpreting the child’s behavior, further deteriorating any form of attachment in the relationship.

Management of reactive attachment disorder

“Children diagnosed with RAD appear to demonstrate significantly more behavioral problems and psychosocial problems than children without RAD” [22], which can negatively impact their educational potential; therefore, employing appropriate management strategies in children with RAD is necessary for reducing the impact it has on the child’s life.

One of the treatment options available for RAD is attachment base therapies, of which the more commonly used is “holding therapy,” also known as rebirthing or reduction therapy [22]. The holding therapy consists of the child being subjected to prolonged restraint and exposure to noxious stimuli, which will continue until the child ceases to escape from the stimuli after which the child is handed over to the caretaker for attachment. Holding therapies, however, has been cautioned by many health professionals, with one of the reasons being that the child could end up having a physical injury, which is another reason why these therapy sessions could end up prolonging the trauma experienced by those who have histories of severe abuse or neglect [22].

Another pathway of treatment that can be utilized falls under the category of “empirically supported treatments”; these treatments “primarily focus on helping parents improve the quality of interactions with their child, communicate behavioral expectations clearly, and provide appropriate consequences for child behavior.” Behavior management training (BMT) is one of the treatment avenues that proved to be promising in decreasing problematic behaviors in children. BMT is developed in such a way that it aids caregivers with “psychoeducation about childhood misbehavior as well as instructs caregivers on parenting skills they can use to increase compliance, decrease disruptive behavior, establish proper disciplinary systems, and improve school behavior with a home-based reward system.” It was found to have lower termination rates and to be successful in reducing problematic behavior in children between 6 and 11 years of age 18. BMT was also found to have better outcomes when compared to attachment-based therapies in terms of it having a time limit, being goal and behaviorally directed, and involving the participation of the parent [22].

Pharmacological intervention in the treatment of RAD in children with other comorbid conditions was observed in a case study conducted by Weinberg in 2010 [13]. Two cases that the study looked at were both children diagnosed with disinhibited RAD associated with other comorbidities; both children were started on a regimen of sertraline and both showed improvement in their moods and behavior at home and in school [13]. Wismer et al. noted that “children with an early history of severe maltreatment have shown reduced levels of vasopressin during an experimental social physical interaction study with their adoptive mothers when these levels are expected to rise” [23]. The reduction in vasopressin and even oxytocin can be implicated in the lack of development of social and emotional bonds in children exposed to maltreatment.

Bosmans et al. [24] focused on the special educational needs of children with RAD. The study confirmed the importance of including teachers in the treatment of children with RAD; it suggested that interpersonal relationships within the class help children in having emotionally healthy interactions with individuals. Most of the studies concluded that effective strategies in dealing with children of different ages may differ and hence there is a need for age-specific intervention programs; examples of interventions include video-feedback intervention to promote positive parenting and sensitive discipline [VIPP-SD], middle childhood attachment-focused therapy, and attachment-based family therapy. The purpose of these programs is to help caregivers discern the unusual signs in the behavior of a child typically stemming from underlying attachment needs. Attachment and bio-behavioral catch-up is another intervention developed to help caregivers understand their child holistically, develop strategies to counteract automatic responses to behaviors attributable to RAD, and overall aid parents in responding to a child’s basic needs. Dyadic developmental psychotherapy provides a safe and controlled environment for the child to express any negative relational experiences with their caregivers, which can help restore the child’s trust in the caregiver. It helps improve attachment relationships in children who encountered trauma during their early years [24]. All the interventions noted have a significant gap between the literature and research conducted, and therefore much more research needs to be carried out.

Morbidity of reactive attachment disorder

An epidemiological study conducted by Minnis et al. focused on the prevalence of RAD in the general population and found a prevalence of 1.4% [25]. Another study conducted by Lehmann et al. in 2013 found that the prevalence of RAD in a group of 391 children between 6 and 12 years of age was around 19.4% [26]. In a study by Hong et al., “the annual diagnosis incidence of RAD per 100,000 population was 5.25-5.39 in 2010, 4.90 in 2011, and 5.45 in 2012. Every year, the incidence was the highest for children aged 2-3 years and showed a decreasing trend with increasing age. The same study also noted that there were severe limitations in the evidence-based epidemiological studies of RAD.

Hong et al. [27] examined the medical and psychiatric comorbidities associated with RAD and concluded that language disorders proved to be one of the common comorbidities in both boys and girls aged 0-3 and 4-6 years, while ADHD was commonly present in both sexes in the ages of 7-9 years. Organically, respiratory system disorders were the most common comorbidity identified in patients diagnosed with RAD [27]. Children in the study in the ages of 4-6 years were found to have higher rates of comorbidities when compared to those aged 0-3 and 7-9 years, the comorbidities observed were “for mental retardation, behavioral and emotional disorders, ADHD, anxiety disorder, affective disorder, behavioral syndromes associated with physiological disturbances and physical factors, schizophrenia, unspecified mental disorder, and enuresis and encopresis.” However, as far as conduct disorders, reaction to severe stress, adjustment disorder, and tic disorders were concerned, children aged 7-9 years had higher comorbidities when compared to the other two age groups. Non-psychiatric symptoms in RAD patients showed no obvious differences in trends concerning age and sex, although RAD patients had higher comorbidity rates for “physical diseases such as skin, infectious and genetic diseases” [27]. A study by Ogundele in 2018 described some potential early symptoms including failure to gain weight or feeding difficulties developing into unusual eating habits, and lack of empathy or impulse control, which could lead to criminal behaviors and cruelty to animals as the child grows older [28]. Pritchett et al. carried out an epidemiological study involving 1,600 children and concluded that RAD was present in the general population and could be a result of both environmental and genetic factors. Children who have compromised lives or disrupted attachments early on in their development are at a significant risk of subsequent developmental difficulties and will then require specialist children's services [1].

## Conclusions

RAD is an under-recognized condition that has a profound impact on the psychological and even organic well-being of children. Identifying risk factors such as institutionalized care, prior history of any form of child abuse, and even poverty can lead to adequate management and prevent severe neurobiological changes that quite closely resemble other psychiatric disorders. It is imperative to adequately help children who may have this disorder or the underlying risk factors by referring them to an appropriate psychiatric service and aiding them in receiving appropriate interventions and follow-up.
